# Local and Widespread Slow Waves in Stable NREM Sleep: Evidence for Distinct Regulation Mechanisms

**DOI:** 10.3389/fnhum.2018.00248

**Published:** 2018-06-19

**Authors:** Giulio Bernardi, Francesca Siclari, Giacomo Handjaras, Brady A. Riedner, Giulio Tononi

**Affiliations:** ^1^Center for Investigation and Research on Sleep, Centre Hospitalier Universitaire Vaudois (CHUV), Lausanne, Switzerland; ^2^Department of Psychiatry, University of Wisconsin-Madison, Madison, WI, United States; ^3^MoMiLab Unit, IMT School for Advanced Studies Lucca, Lucca, Italy

**Keywords:** high-density EEG, slow waves, slow wave activity, NREM sleep, K-complex

## Abstract

Previous work showed that two types of slow waves are temporally dissociated during the transition to sleep: widespread, large and steep slow waves predominate early in the falling asleep period (*type I*), while smaller, more circumscribed slow waves become more prevalent later (*type II*). Here, we studied the possible occurrence of these two types of slow waves in stable non-REM (NREM) sleep and explored potential differences in their regulation. A heuristic approach based on slow wave synchronization efficiency was developed and applied to high-density electroencephalographic (EEG) recordings collected during consolidated NREM sleep to identify the potential *type I* and *type II* slow waves. Slow waves with characteristics compatible with those previously described for *type I* and *type II* were identified in stable NREM sleep. Importantly, these slow waves underwent opposite changes across the night, with only *type II* slow waves displaying a clear homeostatic regulation. In addition, we showed that the occurrence of *type I* slow waves was often followed by larger *type II slow waves*, whereas the occurrence of *type II* slow waves was usually followed by smaller *type I* waves. Finally, *type II* slow waves were associated with a relative increase in spindle activity, while *type I* slow waves triggered periods of high-frequency activity. Our results provide evidence for the existence of two distinct slow wave synchronization processes that underlie two different types of slow waves. These slow waves may have different functional roles and mark partially distinct “micro-states” of the sleeping brain.

## Introduction

During non-REM (NREM) sleep, thalamocortical neurons manifest a strong propensity to fall into a silent, hyperpolarized “*down-state*” after a period of activation (Steriade et al., 1993a, 2001), giving rise to slow waves (0.5–4.5 Hz) in the electroencephalographic (EEG) signal. When cortical neurons are in this “*bistable*” condition, widespread *down-states* can be triggered by a variety of stimuli, including auditory tones and direct cortical activation by transcranial magnetic stimulation (TMS; Massimini et al., 2007; Riedner et al., 2011). Slow wave parameters such as amplitude, slope and number of negative peaks are thought to depend on the number of recruited neurons and the speed of synchronization across neuronal populations (Esser et al., 2007; Riedner et al., 2007; Vyazovskiy et al., 2007). In particular, steep, high-amplitude slow waves with few negative peaks are generated when an efficient neuronal synchronization allows for a fast recruitment of a large number of neurons at the cortical level. Also, synchronization efficiency for typical slow waves tends to be higher at the beginning of the night, when sleep pressure is stronger, and to decline progressively in the course of sleep (Riedner et al., 2007; Vyazovskiy et al., 2007). Such changes are compatible with a net increase of average synaptic strength during wake and a corresponding decrease during sleep (Tononi and Cirelli, 2006, 2014; de Vivo et al., 2017).

We recently showed that during the transition to sleep, the time course, topographic distribution and morphology of slow waves is best accounted for by two distinct neural synchronization mechanisms (Siclari et al., 2014; Spiess et al., 2018). Specifically, the initial part of the falling asleep process is characterized by the emergence, on a relatively flat background, of large and steep slow waves that originate in sensory-motor areas and medial parietal cortex and involve a broad fronto-medial region: we named these waves “*type I” slow waves*. On the other hand, as sleep gets deeper, smaller, shallower *“type II”* slow waves that can originate anywhere in the cortex and involve more circumscribed cortical areas become predominant. These observations suggest that *type I* slow waves may reflect a particularly fast and efficient neuronal synchronization. We proposed that the generation of these slow waves may depend on a “bottom-up,” subcortico-cortical synchronization mediated by arousal-promoting structures. This is because an input from diffusely projecting subcortical sources should trigger a near-simultaneous depolarization in widespread regions of the cortex, followed by a massive hyperpolarization (*down-state*) and by the consequent appearance of large negative waves in the EEG signal (Bellesi et al., 2014). Also consistent with this interpretation, the cortical areas involved by *type I* slow waves are a main target of arousal-promoting neuromodulatory systems (Gaspar et al., 1989; Javoy-Agid et al., 1989; Lewis and Morrison, 1989). EEG oscillations classically identified as *K-complexes* (KC) can also be considered as *type I* slow waves (Colrain, 2005; Halász, 2005; Cash et al., 2009). By contrast, we proposed that *type II* slow waves likely result from a less efficient synchronization process, probably reflecting a “horizontal,” cortico-cortical spreading of a *down-state* originating within a local cortical region.

Importantly, our previous investigation has left open the question whether the distinction between *type I* and *type II* slow waves is limited to the falling asleep period or also maintained during stable sleep. Moreover, if *type I* and *type II* slow waves are organized by distinct synchronization processes, they may also be regulated differently and may differently “interact” with cortical brain activity. In order to answer these questions, we first developed and validated a heuristic classification approach for the identification of widespread (*type I)* and local (*type II)* slow waves throughout sleep. This method was used to prove the existence of slow waves with features compatible with those of *type I* and *type II* slow waves during consolidated NREM sleep. Additional analyses were then performed to investigate how the two types of slow waves are regulated with respect to homeostatic mechanisms and how they interact with ongoing brain activity. We hypothesized that *type I* slow waves, as opposed to *type II slow waves*, would be largely independent from homeostatic changes in sleep pressure, in light of the proposed subcortico-cortical synchronization mechanism. Moreover, we predicted that *type I* and *type II* slow waves would show characteristics that are compatible, respectively, with a single subcortical synchronization system and with multiple independent cortical generators.

## Materials and Methods

### Subjects

Ten healthy volunteers (age 25.4 ± 4.7 years, range 20–34; six males) screened for neurological, psychiatric and sleep disorders and who were not on psychotropic medication underwent an overnight EEG recording in the sleep laboratory. All the subjects reported good sleep quality and scored <10 points on the Epworth Sleepiness Scale (Johns, 1991). This study was carried out under a research protocol approved by the Health Sciences Institutional Review Board of the University of Wisconsin-Madison. All subjects gave written informed consent in accordance with the Declaration of Helsinki.

### Data Acquisition

One overnight high-density (hd-)EEG recording (256 channels; Electrical Geodesics Inc., Eugene, OR, USA) was obtained for each of the 10 participants in a specialized laboratory setting. All recordings were initiated at the usual bedtime of each participant, and interrupted after ~8 h. The EEG signal was sampled at 500 Hz. Sleep scoring was performed over 30 s epochs according to standard criteria by a board-certified physician specialized in sleep medicine (Iber, 2007).

### Data Preprocessing

Each EEG recording was band-pass filtered (0.5–58 Hz), and all 30 s epochs including sleep stages N2 and N3 were extracted. Electrodes containing clear artifacts were visually identified, rejected and replaced with data interpolated from nearby channels using spherical splines. An Independent Component Analysis (ICA) procedure was also used to reduce ocular, muscular, and electrocardiograph artifacts using EEGLAB routines (Delorme and Makeig, 2004; Delorme et al., 2011). After exclusion of 72 electrodes located on the neck/face region (more commonly affected by physiological artifacts), the remaining 185 “internal” channels were retained for further analysis (Massimini et al., 2004; Riedner et al., 2007).

### Slow Wave Detection Procedure

**Figure 1:**
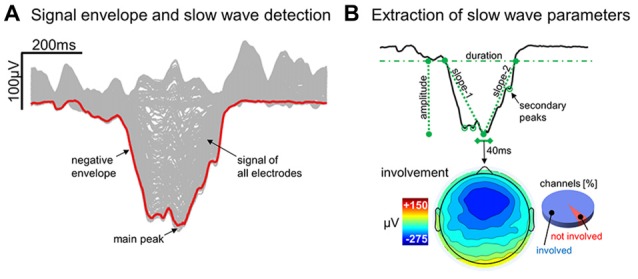
Detection and characterization of slow waves. Panel **(A)** shows the signal corresponding to a representative (type I) slow wave for all individual electrodes (gray lines) and for the negative envelope (red line) computed across all channels (the latter is the signal actually used for slow wave detection). Panel **(B)** depicts the different parameters extracted from the negative envelope of the same slow wave, including duration, amplitude, slope-1, slope-2, number negative peaks and the proportion of involved electrodes.

An automated slow wave detection algorithm similar to the one described in Riedner et al. (2007) was applied to a composite EEG-signal generated from linked-mastoid referenced channels. Specifically, a *negative-going signal envelope* was calculated by selecting the fifth most negative sample across all selected channels (Siclari et al., 2014; Bernardi et al., 2016; the four most negative electrodes were discarded to avoid including potential residual high amplitude oscillations of artifactual origin). The resulting signal underwent broadband filtering (0.5–40 Hz, stop-band at 0.1 Hz and 60 Hz) and a detection procedure based on zero-crossings of half-waves was applied (Riedner et al., 2007). This approach allows to detect both local and widespread slow waves, and to define a unique time reference (across electrodes) for each negative oscillation (Mensen et al., 2016). On the other hand, it should be noted that the shape of a waveform detected on the negative signal envelope does not necessarily correspond to any particular channel (Figure 1A; Mensen et al., 2016). For all the detected slow waves, various parameters of interest were calculated (Figure 1B) and stored for subsequent evaluation, including: duration (time between zero-crossings [s]), amplitude of the maximum negative-peak [μV], number of negative peaks, slope-1 (between the first zero-crossing and the maximum negative peak [μV/s]), slope-2 (between the maximum negative peak and the second zero-crossing [μV/s]), and involvement (mean EEG signal calculated across all electrodes in a 40 ms window centered on the wave peak [μV]). Only negative half-waves with a duration between 0.25 s and 1.0 s were selected for subsequent analyses (full-wave period 0.5–2.0 s). The maximum length was specifically selected to avoid the potential inclusion of residual artifacts, related for instance to slow eye movements or sweating, which are known to affect frequencies around and below 0.5 Hz. In order to accurately detect homeostatic changes in slow wave characteristics, no amplitude threshold was applied (Riedner et al., 2007; see Supplementary Text and Supplementary Figure S1 for a comparison with slow wave detection approaches adopted in previous work; Siclari et al., 2014).

### Classification of *Type I* and *Type II* Slow Waves

Previous results obtained from data collected at sleep onset have suggested the existence of a temporal dissociation between two distinct synchronization processes during the transition to sleep (Siclari et al., 2014). Specifically, we showed that early epochs of the falling asleep process are characterized by large and steep “*type I*” slow waves, emerging on a relatively flat background, while late epochs show a net predominance of smaller “*type II*” slow waves (Supplementary Text and Supplementary Figure S1). Moreover, early (*type I)* slow waves displayed properties reflecting a strong efficiency in neuronal synchronization (such as a steep slope and large amplitude; Esser et al., 2007), while late (*type II)* slow waves, displayed properties indicating a slower and more localized recruitment of neuronal populations into the *down-state* (Siclari et al., 2014). These observations imply that *type I* and *type II* slow waves could be defined based on their synchronization strength. Thus, in the present study, we defined a slow wave classification approach based on the calculation of a “*synchronization score”* (SS). This parameter was defined as the *relative scalp involvement—*expressed as the percentage of channels showing a negative averaged current value of <−5 μV in the 40 ms time-window centered on the reference wave peak—multiplied by the wave *mean slope* (i.e., the mean of slope-1 and slope-2 [μV/ms]). Thus, the SS should reflect both the amount of “*cortical units*” (i.e., neurons) contributing to the EEG slow wave (scalp involvement) and the rapidity of their synchronization (slope; Esser et al., 2007).

**Figure 2:**
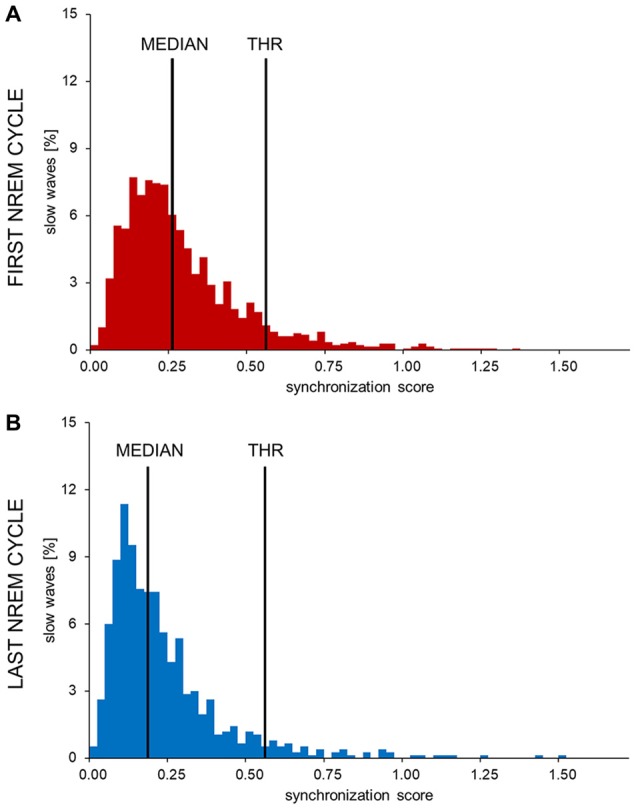
Histograms showing the distribution of synchronization scores (SS) in one representative subject. The SSs observed in the first **(A)** and last **(B)** sleep cycle were divided in 0.025 SS-units bins to obtain these images. The y-axis shows the percentage of all detected waves in each bin. In all subjects and examined conditions the SS showed a non-normal, skewed distribution. The long, sparse, right tail of the distribution is compatible with the presence of highly synchronized slow waves. An arbitrary threshold to distinguish between potential type I and type II slow waves (THR) was set at three median absolute deviations (MAD) from the median of the early SS distribution.

The distribution of synchronization scores (SS) for slow waves detected during the first NREM cycle (Figure 2) appeared as non-Gaussian and was characterized by a positive skewness. While a clear bimodal distribution of the SS was not identifiable, high SS slow waves appeared to be fewer in number and displayed a more variable synchronization strength compared to low SS slow waves. Of note, a re-analysis of data collected at sleep onset using this method yielded a very similar distribution, as shown in Supplementary Figure S2. Based on these observations, a putative distinction between *type I* and *type II* slow waves was made by defining an arbitrary SS threshold corresponding to the median of the distribution plus three times the median absolute deviation (MAD; Figure 2). Slow waves characterized by a SS greater than the set threshold (tail of the distribution) were defined as *type I* slow waves, while remaining slow waves (main body of the distribution) were classified as *type II slow waves*. The threshold was calculated on the SS distribution of slow waves detected during the first NREM cycle (if not stated otherwise, analyses were focused on this specific cycle to avoid potential confounds related to relative overnight changes in slow wave characteristics).

### *Type I* and *Type II* Slow Waves in Stable NREM Sleep

Several analyses have been performed to verify whether properties of slow waves classified as *type I* or *type II* using the heuristic approach described above were consistent with those previously observed based on the temporal dissociation during the falling asleep process (also see Supplementary Figure S3; Siclari et al., 2014). In particular, we based our analyses on the observation that slow waves occurring early in the falling asleep period (including many *type I)*, relative to those occurring later (prevalently *type II*): (i) are typically larger, steeper and characterized by a lower number of negative peaks (*morphological criterion*); (ii) tend to mainly involve frontal areas and often “spare” posterior brain regions (*involvement criterion*); and (iii) mainly originate in a specific region encompassing the sensorimotor cortex and medial parietal areas (*origin criterion*).

#### Morphology

Average properties of NREM slow waves classified as *type I* or *type II* were calculated and compared qualitatively with those reported in previous study. Examined parameters included: amplitude, slope-1, slope-2, duration and number of negative peaks.

#### Involvement

The scalp involvement of NREM slow waves classified as *type I* or *type II* was calculated, z-scored transformed across electrodes (to allow the direct comparison of slow waves with very different amplitudes), and averaged within each subject. Regional differences were investigated using group-level paired *t-tests* at each channel location. A complimentary evaluation based on Principal Component Analysis (PCA) of scalp involvement was also used, as described in Supplementary Material (Supplementary Table S1 and Supplementary Figure S4).

#### Origin

For each slow wave, the pattern of scalp-level propagation was calculated using an approach similar to those described in other recent works (Massimini et al., 2004; Menicucci et al., 2009; Mensen et al., 2016). Briefly, for each electrode, the latency of the negative peak (if present) was calculated with respect to the timing of the main negative peak of the wave detected on the signal negative envelope, and a “delay-map” was computed for each slow wave (see Supplementary Text for detailed description). For this particular analysis, only slow waves characterized by a minimum propagation time of 20 ms were further analyzed. This threshold was applied to ensure inclusion of a sufficient number of slow waves with well-recognizable propagation pattern (similar results were obtained with a 10 ms or a 30 ms threshold; *data not shown*). The “origin” of a slow wave was defined as the geometric centroid (center of mass) of the group of electrodes showing a maximum delay of 5 ms (this value was preferred over a “0 ms latency” to take into account potential inaccuracies in the peak latency estimation).

### Homeostatic Changes in *Type I* and *Type II* Slow Waves

If, as previously hypothesized, *type I* slow waves reflect a subcortico-cortical, rather than a cortico-cortical synchronization mechanism, their properties could be expected to be largely independent from homeostatic changes related to variations in sleep pressure. In fact, such changes have been linked to variations in synaptic strength and cortico-cortical interaction (Tononi and Cirelli, 2014), and should therefore mainly affect slow waves characterize by a predominant cortico-cortical synchronization (*type II*).

In order to evaluate the effect of homeostatic changes occurring during a night of sleep, *type I* and *type II* slow waves detected during the first and last NREM cycle were directly compared with respect to amplitude, slope-1, slope-2, duration and number of negative peaks. A first vs. last cycle comparison was chosen (vs. a split-night approach; e.g., Marzano et al., 2010) to maximize potential homeostatic differences in slow wave parameters (e.g., Riedner et al., 2007) and thus detect even relatively small modifications.

### Relationship Between Background Brain Activity and Synchronization Efficiency

In line with the differential synchronization mechanisms hypothesized for *type I* and *type II* slow waves, one could expect properties of *type II* (but not *type I*) slow waves to show a tight relationship with the relative level of cortical *bistability*, potentially influencing both the frequency of spontaneous *down-states* and the efficiency of their synchronization.

To evaluate how brain activity immediately preceding the appearance of a slow wave influences the amplitude of the subsequent *type I* or *type II* slow wave, 2 s of EEG activity preceding a reference point placed 500 ms prior to the first wave zero-crossing were extracted from two small regions of interest (ROIs) centered on channels Fz and Pz (these channels were selected because they have been shown to be more frequently involved in *type I* and *type II* slow waves, respectively). The pre-wave gap of 500 ms was added to exclude positive signal deflections preceding the negative half-wave, and to minimize the impact of oscillations (e.g., spindles) potentially accompanying the slow wave (e.g., Steriade, 2006; Siclari et al., 2014; Yordanova et al., 2017). The power spectral density was calculated for the 2-s EEG segments using the Welch’s method in Hamming windows (eight sections, 50% overlap, 0.5 Hz bin resolution). Adjacent frequency bins were integrated to obtain a 1 Hz resolution in the 1–30 Hz range. For each subject, the correlation between the power spectral density in each frequency bin and the amplitude of the following slow wave was calculated.

In addition, to evaluate how *type I* slow waves influence *type II* slow waves and vice versa, the temporal distance between slow waves of the same and different type and the proportion of slow waves occurring in close association was calculated. Two waves were defined as “associated” if the distance between the end of the preceding half-wave and the beginning of the following *down-state* was <1 s. We then identified all *type I* and *type II* slow waves occurring in association with a wave of the same or different type, and determined the potential effect of this association on the slow wave synchronization by comparing the amplitude of slow waves preceded by another slow wave and those showing no association (inter-wave distance >4 s).

### Changes in Brain Activity Related to the Occurrence of *Type I* or *Type II* Slow Waves

Given that *type I* and *type II* slow waves may have different generation mechanisms (Siclari et al., 2014), we hypothesized that these slow waves would be associated with distinctive changes in cortical activity. For instance, the involvement of the arousal system in the synchronization of *type I* waves could be expected to lead to a temporary increase in excitatory neuromodulation and to consequent changes in high-frequency activity.

To compare changes in brain activity following *type I* or *type II* slow waves, we proceeded as follows: for each slow wave detected on the signal negative envelope, we identified two 6 s time-windows, one ending 1 s prior to the first zero-crossing (positive-to-negative), the other one starting 1 s after the second zero-crossing (negative-to-positive). The duration of 6 s was selected to identify relatively sustained changes in brain activity, and this length was preferred over longer periods to avoid the possible dissipation of induced changes as a function of time (e.g., see Halász, 1993). The 1 s gap was introduced to avoid including increases in spindle or gamma activity during the down-to-up-state transition (Steriade, 2006; Valderrama et al., 2012). For each 6 s window we calculated the signal power within 1 Hz bins (1–30 Hz range) in seven fronto-central electrodes (around and including Fz). Pre-wave and post-wave power values were averaged across electrodes and log-transformed. Then, for each frequency bin, the % power variation from pre-wave to post-wave was calculated. Brain activity variations triggered by *type I* and *type II* slow waves were directly compared to identify potential differences.

### Statistical Analyses

If not stated otherwise, statistical comparisons described in present work were performed using two-sided paired *t*-tests at group-level (*N* = 10; *p* < 0.05). When multiple tests were performed on related hypotheses, a Bonferroni adjustment was applied to ensure correction for multiple comparisons.

## Results

### *Type I* and *Type II* Slow Waves in Stable NREM Sleep

**Figure 3:**
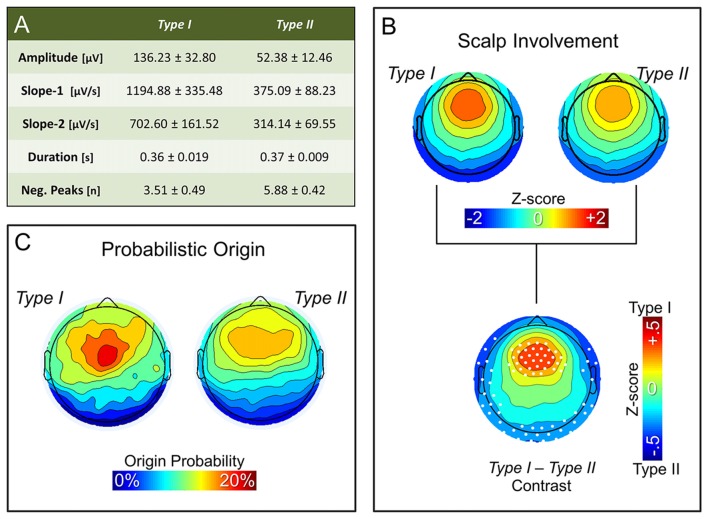
Properties of non-REM (NREM) slow waves classified as type I and type II. Panel **(A)** displays the mean ± SD for the main morphological properties of slow waves detected during the first NREM cycle: amplitude, slope-1, slope-2, duration and number of negative peaks. Topographic plots in panel **(B)** represent the mean scalp involvement of type I and type II waves, as well as the statistical comparison between the two (*p* < 0.05, Bonferroni corrected). Finally, topographic plots in panel **(C)** show the distribution of the probabilistic origin for the two types of slow waves.

Figure 3A summarizes the characteristics of *type I* and *type II* slow waves identified during the first NREM sleep cycle. As expected, features of slow waves classified as *type I* and *type II* using the synchronization score (SS) were consistent with those previously described based on the temporal dissociation across early and late epochs of the falling asleep period (also see Supplementary Figure S3A): *type I* slow waves had larger amplitude, steeper slopes and less negative peaks, as compared to *type II* slow waves (similar results were obtained for the last NREM cycle; *data not shown*). Of note, some of these features (amplitude and slopes) are directly related to properties that were used for calculation of the *synchronization score*. However, consistency with previous findings is observed also for the number of negative peaks, a feature previously suggested to have an inverse relationship to synchronization efficiency (Esser et al., 2007; Riedner et al., 2007).

Additional analyses also confirmed that NREM slow waves classified as *type I* tend to have a stronger involvement of fronto-central brain areas (Figure 3B; Supplementary Figures S3B, S4), and have a preferred origin hotspot under central electrodes, potentially covering the sensori-motor cortex (Figure 3C). *Type II* slow waves, on the other hand, more often involve posterior brain regions (Figure 3B; Supplementary Figure S3C) and display a tendency to originate from different locations all over the cortex (Figure 3C), with a relative preference for medial and lateral centro-frontal areas.

### Changes in Properties of *Type I* and *Type II* Slow Waves in the Course of the Night

**Figure 4:**
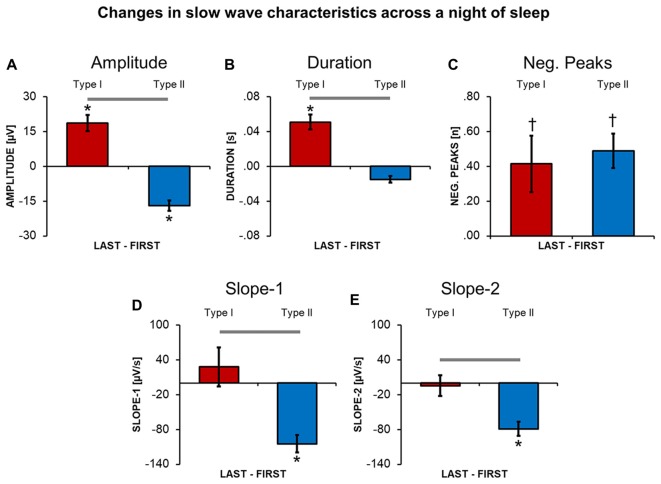
Changes in slow wave characteristics throughout a night of continuous sleep. Each barplot displays the difference between the last and the first NREM sleep cycle for slow waves classified as type I or type II, in: amplitude **(A)**, duration **(B)**, number of negative peaks **(C)**, slope-1 **(D)**, slope-2 **(E)**. Vertical bars indicate standard errors (SE). Horizontal gray bars mark significant differences in relative overnight changes between type I and type II waves. Asterisks mark significant overnight changes for each slow wave type separately. *Bonferroni corrected *p* < 0.05; ^†^uncorrected *p* < 0.05.

A direct comparison between slow waves detected during the first and the last NREM cycle revealed that *type I* and *type II* slow waves undergo distinct changes (Figure 4) in the course of the night. In fact, from the beginning to the end of a night of sleep, *type I* slow waves tended to become larger and to last longer (*p* < 0.05). On the other hand, *type II* slow waves became smaller in amplitude and showed a flatter slope-1 and slope-2. For both *type I* and *type II* slow waves, we observed a non-significant trend (*p* < 0.05, *uncorrected*) towards an increase in the number of negative peaks from the beginning to the end of the night. Overall, these changes are consistent with a relative decrease in slow wave synchronization efficiency for *type II* slow waves only.

### Relationship Between Background EEG Activity and Synchronization Efficiency

**Figure 5:**
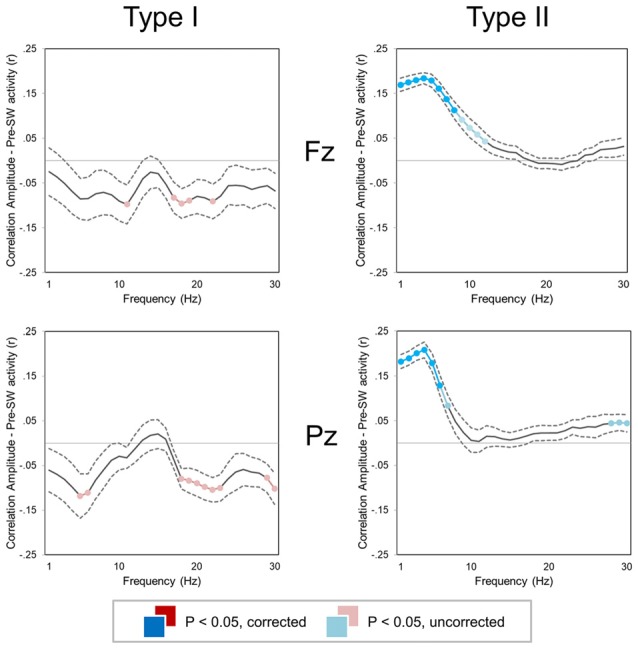
Correlation between slow wave amplitude and background EEG activity (±SE, dashed lines) for two small regions of interest (ROIs) centered on Fz (top) and Pz (bottom). Colored (red, cyan) sections indicate significant correlations (Bonferroni corrected *p* < 0.05; lighter colors indicate uncorrected *p* < 0.05). For both type I (left column) and type II (right column) slow waves, the mean power was computed in 1 Hz frequency bins for the time window corresponding to −2.5 to −0.5 s with respect to the first zero-crossing of the slow wave.

A correlation analysis revealed that amplitude of *type I* and *type II* slow waves was differentially modulated by background EEG activity (Figure 5). In particular, low-frequency power (1–10 Hz) was positively correlated with the amplitude of subsequent *type II*, but not of *type I* slow waves (*p* < 0.05). Indeed, both low-frequency (<12 Hz) and high-frequency (>18 Hz) activity actually showed a trend toward a negative correlation with the amplitude of *type I* slow waves (*p* < 0.05, *uncorrected*). These results suggest that low-frequency and high-frequency oscillations may interfere with the synchronization efficiency of *type I* slow waves and instead favor the appearance of relatively large *type II* waves.

### Interactions Between *Type I* and *Type II* Slow Waves

**Figure 6:**
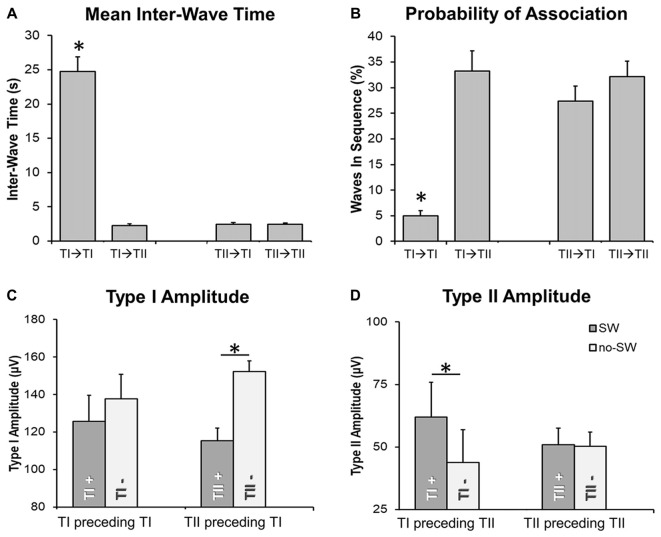
Temporal relationship between slow waves. Barplots in **(A,B)** show that the delay between two type I slow waves is typically longer than the delay between type I and type II slow waves or between two type II slow waves. Barplots in **(C,D)** depict the effect of the “presence” (TI+, TII+; vs. “absence,” marked with TI- or TII-) of a slow wave on the amplitude of a subsequent, associated type I (TI) or type II (TII) slow wave. Two waves were defined as associated if the distance between the end of the preceding half-wave and the beginning of the following negative deflection was <1 s. The *marks significant differences at corrected *p* < 0.05 (when placed above a single bar, the *marks difference between a specific condition and all other examined conditions). Vertical bars indicate SE.

An analysis of the inter-wave interval was performed to evaluate the probability of association between slow waves of the same and different type (Figure 6). We observed that the “*type I—type I”* delay was significantly longer than the “*type II—type II”* delay (Figure 6A; *p* < 0.05). Moreover, the inter-wave time between *type II* and *type I* slow waves (“*type II—type I”* or “*type I—type II”*) was similar to the one observed between *type II* slow waves. *Type II* slow waves were more likely to occur in sequence than *type I* slow waves, consistent with the existence of a refractory period after the generation of a *type I* slow wave.

Finally, we investigated the effect of *type I* and *II* slow waves on the amplitude of subsequent slow waves (Figures 6C,D). Results showed that *type II* slow waves had a larger amplitude when they were preceded (within 1 s) by a *type I* slow wave than when they occurred in isolation (inter-wave time >4 s). Vice-versa, *type I* slow waves had a smaller amplitude when they were preceded by a *type II* slow wave than when occurred in isolation. These findings are consistent with results of the correlation analysis (Figure 5), suggesting that the occurrence of low-frequency oscillations may interfere with the synchronization efficiency of a subsequent *type I* wave. On the other hand, the occurrence of a *type I* slow wave seems to favor a more efficient synchronization of subsequent *type II* slow waves.

### Changes in Brain Activity Related to the Occurrence of *Type I* or *Type II* Slow Waves

**Figure 7:**
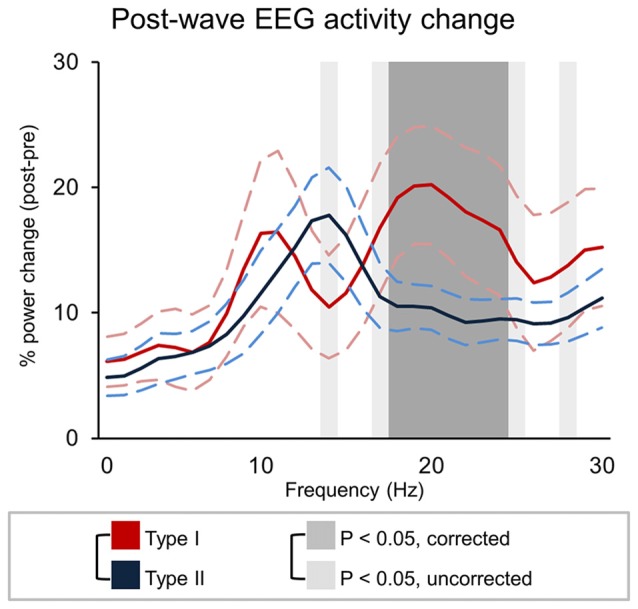
Signal power changes following type I and type II slow waves. The graph show the percent power variation between pre- and post- slow wave periods (±SE, dashed lines). Dark-gray shadows indicate Bonferroni corrected *p* < 0.05, while light gray indicate results at uncorrected *p* < 0.05.

The analysis of changes in brain activity following the occurrence of different types of slow waves revealed that t*ype I* slow waves tend to be followed by two distinct peaks of relative power increase, not clearly present for *type II* waves, in the alpha (~10 Hz; *p* < 0.1, *uncorrected*) and beta (>18 Hz; *p* < 0.05) ranges (Figure 7). Such changes may reflect a specific association with EEG micro-arousals (Iber, 2007). Conversely, *type II* slow waves appeared to induce an isolated increase peak in sigma power (maximum at 14 Hz, in the spindle range; *p* < 0.05, *uncorrected*). A partial frequency-band overlap between alpha (*type I*) and sigma (*type II*) changes likely prevented the attainment of statistically significant differences between *type I* and *type II* slow waves in these ranges.

## Discussion

The present study aimed to investigate whether *type I* and *type II* slow waves, previously distinguished based on their temporal dissociation during the falling asleep process, are also present during stable sleep, and whether they show distinct regulation patterns in line with the involvement of different synchronization mechanisms. We developed and validated a heuristic approach based on synchronization efficiency for classifying *type I* and *type II* slow waves in high-density EEG recordings collected during continuous sleep. We found, first, that slow waves with distinct *type I* and *type II* features occur not only during the wake-sleep transition, but also during stable NREM sleep. Second, we found that *type I* and *type II* slow waves undergo distinct changes from the beginning to the end of a night of sleep. Finally, we found that *type I* and *type II* slow waves show a different modulation depending on preceding brain activity and, in turn, have distinct effects on following brain activity. Altogether, these results support the validity of the distinction between *type I* and *type II* slow waves with respect to their mechanisms of synchronization, homeostatic regulation, and their interactions with neural activity before and after their occurrence.

### *Type I* and *Type II* Slow Waves During Stable NREM Sleep

In the present work, several complementary analyses based on morphological and topographical criteria related to involvement and origin distributions were used to characterize slow waves with high (*type I*) and low (*type II*) levels of synchronization efficiency in consolidated NREM sleep. The results show that the same differences between *type I* and *type II* slow waves that were previously reported between early and late slow waves during the falling asleep period are also presented during consolidated NREM sleep (Siclari et al., 2014). The two types of slow waves identified during the falling asleep process had several distinct properties (Siclari et al., 2014): *type I* slow waves, which are prominent during the initial phase of the transition to sleep, are large and steep, tend to originate in the sensorimotor and medial parietal cortex and mainly involve frontal brain areas, while *type II* slow waves, which are prevalent in the late part of the falling asleep process, are typically smaller and less steep, and tend to have more variable origin and a more posterior cortical distribution. While a clear temporal dissociation was observed during the wake-sleep transition, visual inspection of individual waves revealed that slow waves with properties compatible with those of *type I* slow waves could also be observed toward the end of the falling asleep process (et vice-versa; Siclari et al., 2014), suggesting that the two subtypes of slow waves may coexist during stable sleep. The present study extends these previous findings by showing that slow waves with features of *type I* and *type II* slow waves are not restricted to the falling asleep period, but also occur in stable NREM sleep, defined using standard criteria (Iber, 2007). In this respect, it is important to note that many previous studies aimed at investigating slow wave properties during stable NREM sleep applied automated slow wave detection algorithms with detection criteria likely favoring the identification of high-amplitude, widespread slow waves (Mölle et al., 2002; Massimini et al., 2004). Thus, such approaches may have been biased towards the detection of *type I* slow waves, rather than of smaller, more local *type II* slow waves (Mensen et al., 2016). Our previous and present findings showing that these two types of slow waves have very different properties and regulation emphasize the importance of choosing detection criteria in a way that is appropriate to the questions being asked.

### *Type I* and *Type II* Slow Waves Show Opposite Changes Across a Night of Sleep

Our results show that *type I* and *type II* slow waves not only differ in morphological and topographical features, they also undergo opposite changes across the night. It is now well known that in the course of a night of sleep, slow waves typically display a decrease in amplitude and slope, mirroring the homeostatic decrease in sleep need (Riedner et al., 2007; Vyazovskiy et al., 2007). It has been suggested that these changes may reflect two mechanisms: (i) a reduced oscillation of the membrane potential of individual neurons; and/or (ii) a poorer synchronization between neurons towards the end of the night (Esser et al., 2007; Riedner et al., 2007). Both mechanisms could result from a net reduction in cortical synaptic strength, although variations in neuromodulation and inhibitory tone may also play a role. Our results suggest such a homeostatic regulation for *type II* but not for *type I* slow waves and may contribute to explain previous observations indicating that the largest slow waves at the end of the night can be as large as those detected during the first NREM cycle, despite a clear decrease of the average slow wave amplitude (Riedner et al., 2007). Of note, when considering all slow waves together, the lack of homeostatic decline of *type I* slow waves may be masked by the net prevalence of *type II* slow waves.

Overall, these results are consistent with the possible existence of two distinct synchronization mechanisms: a cortico-cortical synchronization process, which is influenced by the efficiency of regional network synchronization and is homeostatically regulated, potentially reflecting underlying changes in local synaptic strength (Tononi and Cirelli, 2014); and a subcortico-cortical synchronization process that appears to be largely independent of the efficiency of regional circuits. However, the effectiveness of the synchronization mechanism underlying *type I* slow waves is likely to be influenced by both the level of activation of the subcortical generator and the functional state of cortical neuronal populations. In this perspective, the relative increase in the amplitude of *type I* slow waves toward the end of the night may be the consequence of an increased activity of the subcortical arousal system, but may also depend on the decreased number and amplitude of local *type II* slow waves that occur out-of-phase with *type I* slow waves and may limit their spread (see below; Riedner et al., 2007).

### Interactions Between *Type I* and *Type II* Slow Waves and With Ongoing Brain Activity

We found that the amplitude of *type II* slow waves was larger when they occurred during a period characterized by prominent low-frequency power (below 10 Hz). Conversely, *type I* slow waves tended to be smaller when they occurred on a low frequency EEG background. In line with this observation, we showed that *type II slow* waves were larger when they were preceded by a *type I* slow wave. Vice-versa, *type I* waves were smaller when preceded by another slow wave. Overall, these results suggest that the occurrence of widespread, *type I* slow waves may favor the subsequent synchronization of local oscillatory events (*type II* slow waves), possibly through a generalized “resetting” of neural slow oscillations which would allow distinct neuronal populations to be recruited in the cortical spreading of a new *down-state*. Conversely, a state of regional de-synchronization, in which different brain areas are giving rise to small, local (*type II*) slow waves, could interfere with the synchronization of widespread oscillations. Specifically, a subcortico-cortical triggering event may not be able to recruit neuronal populations that are already or were recently involved in a *down-state* (Vyazovskiy et al., 2009), which would lead to the generation of smaller *type I* slow waves. This possibility is consistent with a previous report indicating that evoked KC may have a lower amplitude during deep sleep (Bastien and Campbell, 1994; i.e., when many regional (*type II*) slow waves are present). On the other hand, reduced bistability, reflected in an increase of high-frequency power, may lead to reduced neuronal recruitability and thereby to *type I* slow waves with a lower amplitude (Figure 5).

The analysis of the temporal relationship between *type I* and *type II* slow waves revealed that about 30% of detected *type II* slow waves occurred in rapid sequence with another slow wave, either *type I* or *II* (mean inter-wave time of ~2.5 s). On the other hand, slow waves classified as *type I* only rarely (~5%) occurred in sequence with another slow wave of the same type, and the mean observed inter-wave time was longer (>20 s). These results suggest that the occurrence of a *type I* slow wave may temporarily preclude the appearance of another *type I slow wave*, but not of a *type II* slow wave. This points to the existence of a “*refractory period*” of the subcortico-cortical synchronization process underlying *type I* slow waves.

Finally, we found that *type I* and *type II* slow waves are followed by characteristic changes in brain activity, possibly related to their different generation mechanisms. In fact, *type I* slow waves are often followed by a relative temporary increase in alpha and beta frequency ranges, potentially reflecting an association with EEG micro-arousals (Iber, 2007). *Type II* slow waves, on the other hand, are followed by a relative power increase within the spindle range. Thus, while *type I* slow waves may lead to a temporary reduction in sleep depth, *type II* slow waves may favor sleep maintenance and environmental disconnection (Elton et al., 1997; Dang-Vu et al., 2011; Lecci et al., 2017) by triggering spindles through a cortico-thalamo-cortical interplay (Steriade, 2006; Crunelli and Hughes, 2010; Mak-McCully et al., 2017).

### Relationship With Other Slow Wave Classification Approaches

Several previous studies suggested that slow waves may be classified in different subtypes with partially distinct properties or regulation. The most classical subdivision is certainly the one that distinguishes so called KC (Loomis et al., 1937; Colrain, 2005) from regular slow waves. Importantly, KCs share many features of *type I* slow waves. KCs are typically defined as isolated EEG oscillations characterized by a large negative amplitude (Halász, 2005), a long duration (Bremer et al., 1970), and a maximal involvement over frontal brain areas (Bastien and Campbell, 1992). Moreover, KCs are often induced by sensory stimulations (Davis et al., 1939; Roth et al., 1956; Riedner et al., 2011), are associated with signs of autonomic activation (Pampiglione and Ackner, 1958; Johnson and Karpan, 1968; Heald et al., 1989; Hornyak et al., 1991; Tank et al., 2003), and are occasionally followed by arousals (Colrain, 2005). Finally, it has been suggested that KCs may not show a clear homeostatic density increase after sleep deprivation (Curcio et al., 2003; Sforza et al., 2004) and may have a lower amplitude during deep sleep (Bastien and Campbell, 1994), i.e., when many regional slow waves are identifiable in the EEG. However, the identification of KCs is typically based on the visual detection of particular morphological hallmarks (De Gennaro et al., 2000) that may not be easily recognized, especially in deep sleep or at stage transitions, whereas the criteria for *type I* slow wave detection can be easily integrated into automatic detection procedures. Altogether, it is likely that *type I* slow waves as characterized here include most (and potentially all) classical KCs as well as a fraction of slow waves that do not fulfill all KC hallmarks. Future studies should better investigate the relationship and potential overlap between *type I* waves and KCs.

The Cycling Alternating Pattern (CAP; Parrino et al., 2012), which describes cyclic sequences of long-lasting cerebral activations and deactivations, also recognizes the tight relationship between low-frequency EEG oscillations and arousal-related structures. In fact, CAP activations (A) are classified as: A1, in the presence of pure slow wave components (i.e., no evidence for autonomic arousal), including KCs, slow wave groups or vertex waves; A2, when the slow component is followed by an arousal pattern; A3, consistent with a traditional arousal pattern (Halász, 2016). The A1 pattern is typically more represented during periods of high homeostatic pressure (e.g., first NREM cycle), while the A2 and A3 patterns become more prevalent during periods of lower sleep pressure (Terzano et al., 2005; Halász, 2016) and tend to decrease when the sleep pressure is high, such as after sleep deprivation (De Gennaro et al., 2002). These variations are in part consistent with our observation of opposite changes for *type I* and *type II* slow waves across a night of sleep. However, a direct comparison with CAP analysis is made difficult by at least two limitations: first, A1 patterns include different subtypes of low-frequency oscillations, potentially presenting different features and distinct generation mechanisms (e.g., KCs and slow wave groups); second, the main parameter analyzed in CAP is the incidence of events, which was not evaluated in the present study.

Another classification approach originated from the empirical observation of relative differences between EEG oscillations below and above 1 Hz, which are thought to reflect “*slow oscillations*” (<1 Hz; SO) and “*delta waves*” (>1 Hz) originally described in anesthetized cats (Steriade et al., 1993a,b). Later studies in humans confirmed the existence of several differences between slow waves with frequency peaking below and above 1 Hz. For instance, it was shown that 40 h of prolonged wakefulness lead to a reduction in the density of slow waves below 0.9 Hz and to an increase of slow waves above 1.2 Hz (Bersagliere and Achermann, 2010). Moreover, sleep deprivation was associated with an increased slope of the half-waves and a decreased number of multi-peak waves. In line with these findings, the typical decline in delta activity from the first to the second NREM cycle was not observed at frequencies below 2 Hz (Achermann and Borbély, 1997; but see Campbell et al., 2006). Of note, we previously showed that *type I* waves tend to have a longer duration (lower frequency) relative to *type II* waves (Siclari et al., 2014). However, such a difference was not observed in the present work. This discrepancy may depend on the present use of the negative envelope of the EEG signal for slow wave detection, which may not accurately reflect the actual duration of individual half-waves recorded from different derivations.

In summary, despite the diversity of classification approaches, several studies provide converging evidence regarding the existence of at least two slow wave subtypes in NREM sleep. Moreover, these studies offer support for the conjecture that low-frequency, high-amplitude slow waves may reflect the direct involvement of the arousal system and may escape many of the regulatory mechanisms that instead affect the majority of slow waves.

### Limitations of the Study

In the present study, we adopted a heuristic approach to distinguish between potential *type I* and *type II* slow waves, which was empirically defined based on previously collected evidence (Siclari et al., 2014). Importantly, this approach relies at least in part on the implicit assumption that both the relative proportion and the synchronization efficiency of the two types of slow waves are relatively stable between and within subjects. While several analyses and visual inspection of our data suggest that this assumption may hold true during physiological sleep in healthy individuals, it is not clear how these two types of slow waves interact with other recurring changes in excitability, such as CAPs and ultraslow oscillations (Lecci et al., 2017). Of note, the use of a distribution-based threshold implies that different thresholds may lead to partially different results, by affecting the direction of classification errors (e.g., increasing or reducing the number of slow waves labeled as *type I*). In the present study, the threshold was selected to separate slow waves with high and variable synchronization efficiencies from the main population of relatively uniform slow waves. However, such an approach may not be readily applicable to populations of subjects with altered proportions of different types of slow waves, such as patients with sleep disorders, or in other conditions. Therefore, it will be important to obtain independent criteria for distinguishing the two sub-types of slow waves, for example through the simultaneous assessment of subcortical and cortical activity during sleep. Only in this way will it possible to correctly classify relatively small *type I* waves and relatively large *type II* slow waves based not on their morphological features but on how they are generated. On the other hand, it is clear from our results that our approach successfully distinguished two sub-populations of slow waves, since they showed different and sometimes opposite characteristics in a variety of conditions. More importantly, most of these differences were consistent with the hypothesized distinction between a subcortico-cortical and a cortico-cortical synchronization mechanism underlying *type I* and *type II* slow waves, respectively. At the same time, it should be kept in mind that we only investigated a limited set of time-windows and spatial locations, and future studies will be necessary to better characterize spatial and temporal differences in the regulation of the two types of slow wave.

## Conclusion and Future Directions

Our results provide novel evidence for a differential regulation of local and widespread slow waves, and support the existence of two distinct processes underlying slow wave synchronization during NREM sleep (Siclari et al., 2014). In particular, they are consistent with previous observations suggesting that widespread *type I* slow waves may depend on a subcortico-cortical synchronization mechanism, while a cortico-cortical synchronization process may underlie more local *type II* slow waves. Moreover, our findings suggest that only *synchronization process II*, and not *synchronization process I*, displays a clear homeostatic regulation (Riedner et al., 2007; Vyazovskiy et al., 2007). Widespread and local slow waves may serve different functions in different phases of sleep, and it is conceivable that such functions may be tightly linked to the specific slow wave synchronization mechanism (Vyazovskiy and Harris, 2013; Genzel et al., 2014). In this respect, local *type II* slow waves occurring out-of-phase all over the cortical mantle may contribute to regional processes involved in the regulation of synaptic strength (Tononi and Cirelli, 2006, 2014). Conversely, widespread *type I* slow waves may reflect a regulatory interaction between arousal-related structures and the cortical system. Activating stimuli reaching a highly *bistable* cortico-thalamic system typically trigger a non-differentiated brain response inducing a diffuse functional de-activation that may be “sleep protective” (Massimini et al., 2007). On the other hand, the *down-state* may be followed by “local wakefulness,” reflected in an increased high-frequency activity. Future work should investigate whether the two synchronization processes may be selectively affected in pathological sleep-wake transitions like insomnia and arousal disorders (Schenck, 2014).

## Author Contributions

GB, FS and GT: conceptualization. GB, GH and BR: methodology. GB and FS: investigation and writing—original draft. GB: formal analysis. BR and GT: supervision. GB, FS, GH, BR and GT: writing—review and editing.

## Conflict of Interest Statement

This was not an industry supported study. GT has consulted for Philips Healthcare who endowed the David P. White Chair in Sleep Medicine, held by GT at the University of Wisconsin-Madison. GT has received research support from Philips Healthcare and has been a symposium speaker for Philips Healthcare and Sanofi. BR receives partial salary support from the Philips Healthcare Grant held by GT. The remaining authors declare that the research was conducted in the absence of any commercial or financial relationships that could be construed as a potential conflict of interest.
