# Supplementary material for: Local and Widespread Slow Waves in Stable NREM Sleep: Evidence for Distinct Regulation Mechanisms
Source: Front Hum Neurosci. 2018 Jun 19;12:248. doi: 10.3389/fnhum.2018.00248 (PMC6018150; doi:10.3389/fnhum.2018.00248)
Supplement: Supplementary file 1 [file Data_Sheet_1.docx]

Supplementary Material

Local and widespread slow waves in stable NREM sleep: Evidence for distinct regulation mechanisms

Giulio Bernardi, Francesca Siclari, Giacomo Handjaras, Brady A. Riedner, Giulio Tononi*

*** Correspondence:** Giulio Tononi: gtononi@wisc.edu

# Supplementary Data

**Two distinct synchronization processes in the transition to sleep.** In light of relative methodological differences involving the slow wave detection procedure between our present and previous work ^1^, we first aimed at evaluating whether our previous observation of two dissociated slow wave synchronization processes during the wake-sleep transition could be replicated using the slow wave detection approach applied in the present study. Thus, we reanalyzed data from our previous work, including a total of 141 wake-sleep transitions across 6 healthy adult participants (23.5±5.9 per subject, length 9.3±2.1 min) ^1^. Each falling asleep segment - from the end of alpha activity (i.e., the cessation of the continuous alpha rhythm typical of wakefulness) to 30 sec after the first slow wave sequence (i.e., more than two successive slow waves with a peak-to-peak amplitude of 75 µV) - was extracted and divided in 10 epochs of equal length. Then, slow waves were detected using the same approach described in main text, and their mean density and amplitude were calculated for each epoch and in each subject.

As previously described, the density of slow waves showed a characteristic course during the wake-sleep transition: in all subjects, an initial rapid increase was followed by a slight decrease or a plateau, and then by a new increase (Figure S1A). On the other hand, amplitude variability (standard deviation of amplitude values) reached a peak at the beginning of the falling asleep period and then decreased in subsequent epochs. The distinct courses of slow wave density and amplitude variability is consistent with the presence of relatively isolated, large-amplitude slow waves in early phases of the falling asleep process, and with a net prevalence of more homogeneous, small-amplitude slow waves in later phases. While the course of changes in slow wave amplitude does not clearly reflect this dissociation in the no-threshold condition (Figure S1A), with a minimum amplitude threshold (e.g., 20 µV or 40 µV; Figure 1S-B/C) the time course of slow wave amplitude becomes similar to the one described for amplitude variability. This observation suggests that amplitude variations in the no-threshold condition may be partially masked by relative changes involving small amplitude slow waves. Overall, these results are consistent with the existence of two distinct types of slow waves in the falling asleep process.

**Principal Component Analysis of slow wave involvement.** A Principal Component Analysis (PCA) was used to determine the most typical EEG involvement associated to *type I* and *type II* slow waves. Specifically, for each subject, we first identified the main components explaining ~95% of the total variance related to slow wave involvement (as computed using the procedure described in main text). The maximal ‘contribution’ of each slow wave to each of the obtained components was evaluated and used to assign slow waves to distinct ‘classes’. Finally, the percentage of *type I* and *type II* slow waves in each class was calculated and compared via paired t-tests.

In all subjects, three main components were identified (Figure S4; Table S1): component A (~69%) was characterized by a distributed involvement peaking over fronto-central areas; component B (~18%) showed a dissociation between a central-posterior and a frontal regional involvement; component C (~7%) presented a dissociation between a left and a right involvement at scalp level. This analysis also revealed that *type I* slow waves tend to have a stereotypical involvement (i.e., were explained by one main topographic component) peaking over centro-frontal areas (component A), while the distribution of *type II* slow waves is more variable (i.e. more evenly represented in all the three main components).

**Calculation of slow wave propagation and origin.** To evaluate the pattern of propagation and the potential origin of detected slow waves, we applied the following procedure. For each channel, we identified the local negative maxima closest in time to the reference peak among those occurring in a 300 ms time-window centered on this time-point. Local negative peaks separated from each other by at least 40 ms were identified on the smoothed EEG signal (5 point moving average). In case of multiple peaks, a threshold corresponding to 75% of the maximum negative peak detected in the same electrode was also applied to exclude small, secondary oscillations. Channels in which the potential peak was smaller than 10% of the amplitude of a ‘*prototype*’ slow wave, defined as the oscillation with the largest negative peak at the reference timing (across all channels), were excluded. A ‘*likeness constraint*’ method, similar to the one described in ^2^ was used to discard channels in which the negative wave was excessively dissimilar from the main traveling oscillation. Specifically, we calculated the cross-correlation between the instantaneous phases (estimated using the Hilbert transform ^3^) of the *prototype* wave and of all other EEG signals in a symmetrical time-window ranging from -150 to +150 ms around the reference peak. The 25^th^ percentile of the distribution of the maximal correlation values (C) was then used as a threshold to exclude events dissimilar from the *prototype* wave. The latency of all remaining local peaks was subsequently used to create a preliminary scalp ‘*delay-map*’. These criteria allowed to exclude potential peaks that were unrelated to the main negative wave propagation, such as deflections of potential artifactual origin or those generated by regional, non-synchronous slow waves. For each preliminary *delay-map*, a *spatio-temporal clusterization* procedure was applied to exclude potential propagation gaps. Local peaks of two spatial neighbor electrodes had to be separated by less than 10 ms in order to be considered as part of the same propagation cluster. Then, the propagation cluster including the prototype wave was identified and the final *delay-map* was extracted. In each map the minimum peak latency was set to 0 ms. Only slow waves characterized by a minimum propagation time of 20 ms were included in subsequent analyses. The ‘*origin*’ of a slow wave was defined as the geometric centroid (center of mass) of the group of electrodes showing a maximum delay of 5 ms (this value was preferred over a ‘0 ms latency’ to take into account potential inaccuracies in the peak latency estimation).

**References**

1. Siclari F, Bernardi G, Riedner BA, LaRocque JJ, Benca RM, Tononi G. Two distinct synchronization processes in the transition to sleep: a high-density electroencephalographic study. Sleep 2014;37:1621-37.

2. Menicucci D, Piarulli A, Debarnot U, d'Ascanio P, Landi A, Gemignani A. Functional structure of spontaneous sleep slow oscillation activity in humans. PLoS One 2009;4:e7601.

3. Cohen L. Time-frequency analysis, 1995.

# Supplementary Figures and Tables

**Table S1**

| **Subject** | **Component A** | **Component B** | **Component C** |
| --- | --- | --- | --- |
| **S01** | 65.73 | 20.19 | 8.14 |
| **S02** | 73.40 | 13.75 | 7.63 |
| **S03** | 70.70 | 17.50 | 5.82 |
| **S04** | 82.05 | 10.11 | 4.28 |
| **S05** | 75.03 | 16.56 | 4.77 |
| **S06** | 78.29 | 9.95 | 7.46 |
| **S07** | 69.69 | 15.64 | 7.73 |
| **S08** | 64.33 | 21.65 | 7.45 |
| **S09** | 65.27 | 19.09 | 8.05 |
| **S10** | 50.14 | 34.86 | 6.55 |
| **Average** | 69.46 | 17.93 | 6.79 |
| **STD** | 8.92 | 7.12 | 1.39 |

***Table S1****. Three main PCA components explain ~95% of the variance associated to slow wave scalp involvement. Component A represents ~69% of the variance; Component B represents ~18% of the total variance; Component C represents ~7% of the total variance.*

**Figure S1**


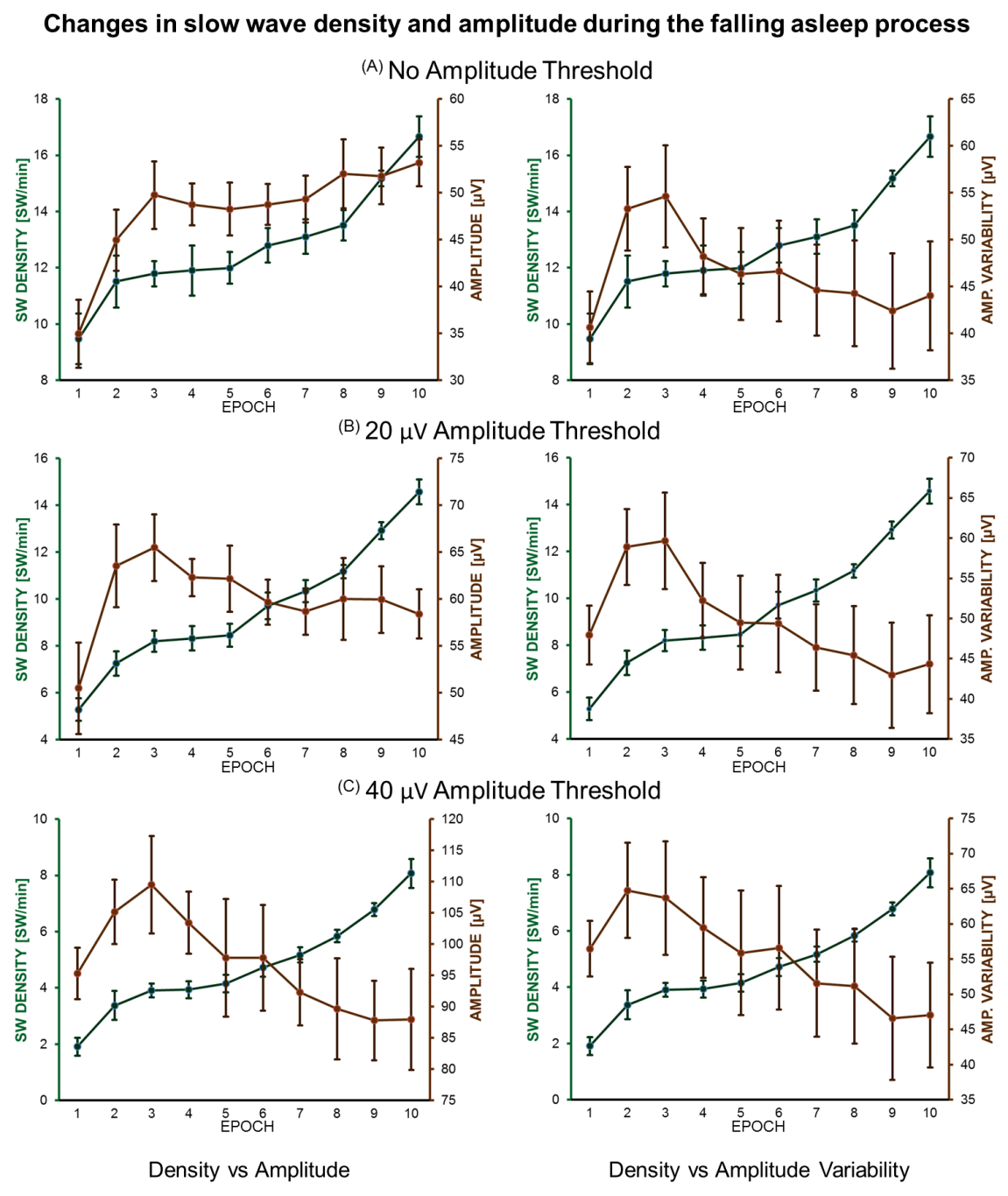


**Figure S1**. *Graphs in the left column compare changes in density (slow waves per minute, SW/min) and in amplitude (µV), while graphs in the right column compare changes in density and in amplitude variability (standard deviation of the amplitude). Vertical bars indicate standard errors (SE). Data extracted from homologous epochs of the wake-sleep transition were averaged as in previous work, and the time course of the parameters of interest was qualitatively evaluated both in single subjects and at group level. Similar graphs are obtained with different negative peak amplitude thresholds, corresponding to 0 µV (A, as for analyses in present work), 20 µV (B) and 40 µV (C).*

**Figure S2**


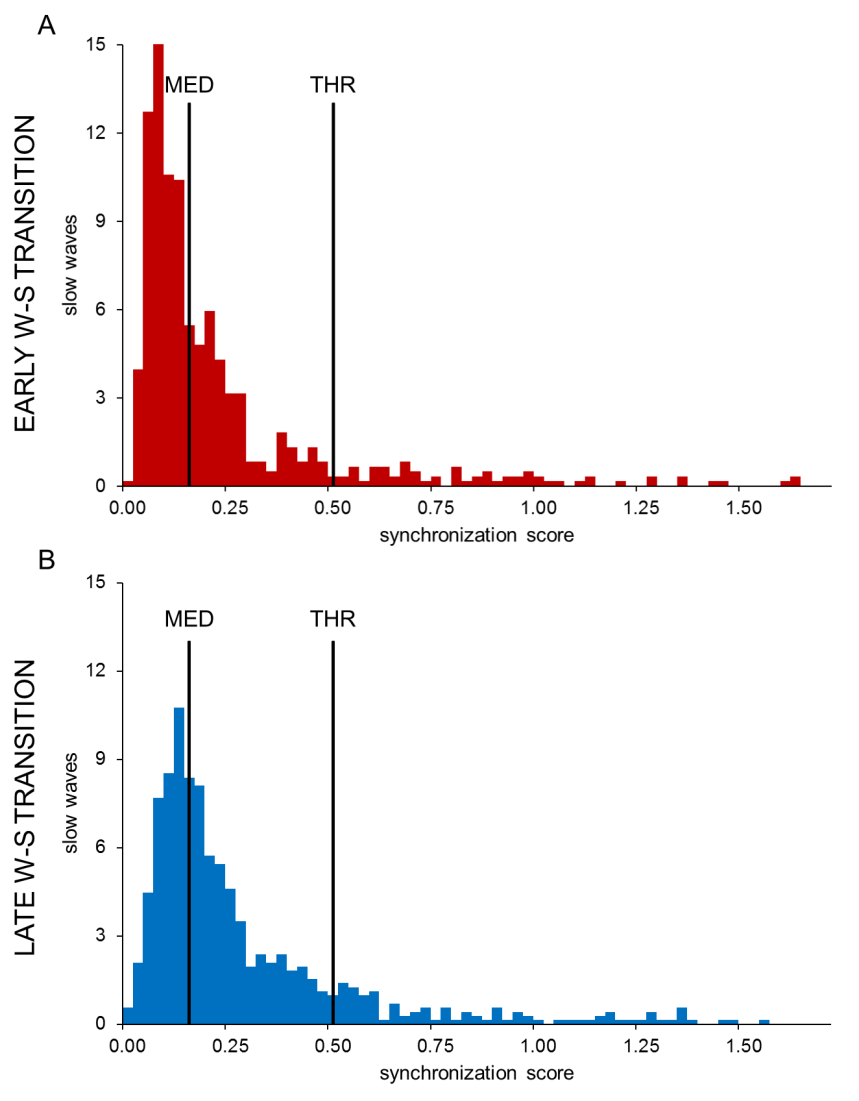


***Figure S2****. Histograms showing the distribution of synchronization scores (SS) in one representative subject. The SS observed in early (A) and late (B) epochs of the transition to sleep were divided in 0.025 SS-units bins to obtain these images. An arbitrary threshold to distinguish between potential type I and type II slow waves was set at 3 MAD (median absolute deviation) from the median of the early SS distribution. Of note, the distributions are similar to those observed during the first and last NREM cycles (Figure 2 in main text).*

**Figure S3**


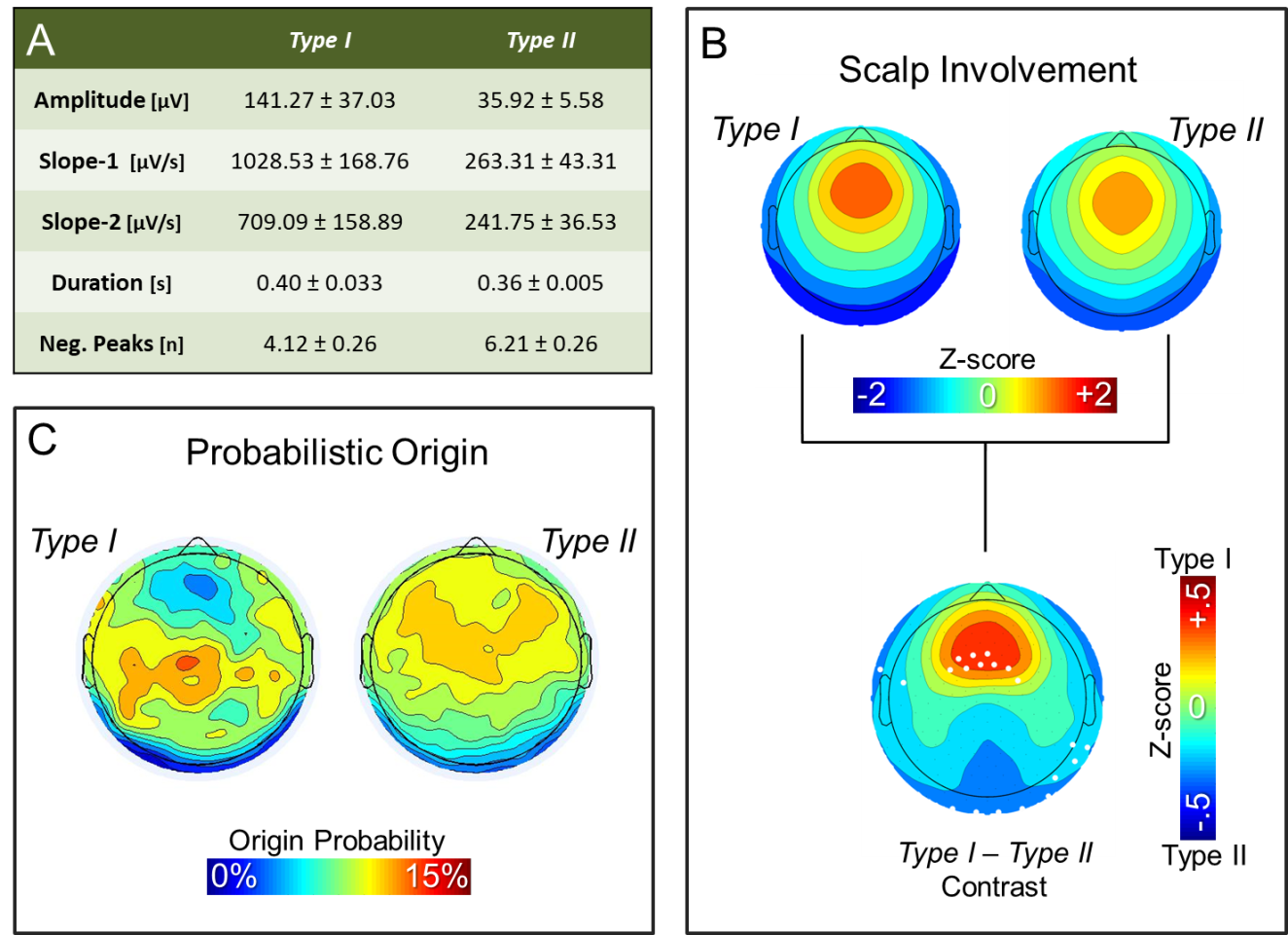


***Figure S3****. Properties of slow waves of the falling asleep period classified as type I and type II (data re-analyzed from previous work). Panel A displays the mean ± SD for the main morphological properties of slow waves: amplitude, slope-1, slope-2, duration and number of negative peaks. Topographic plots in panel B represent the mean scalp involvement of type I and type II waves, as well as the statistical comparison between the two (p < 0.0005, uncorrected). Finally, topographic plots in panel C show the distribution of the probabilistic origin for the two types of slow waves.*

**Figure S4**


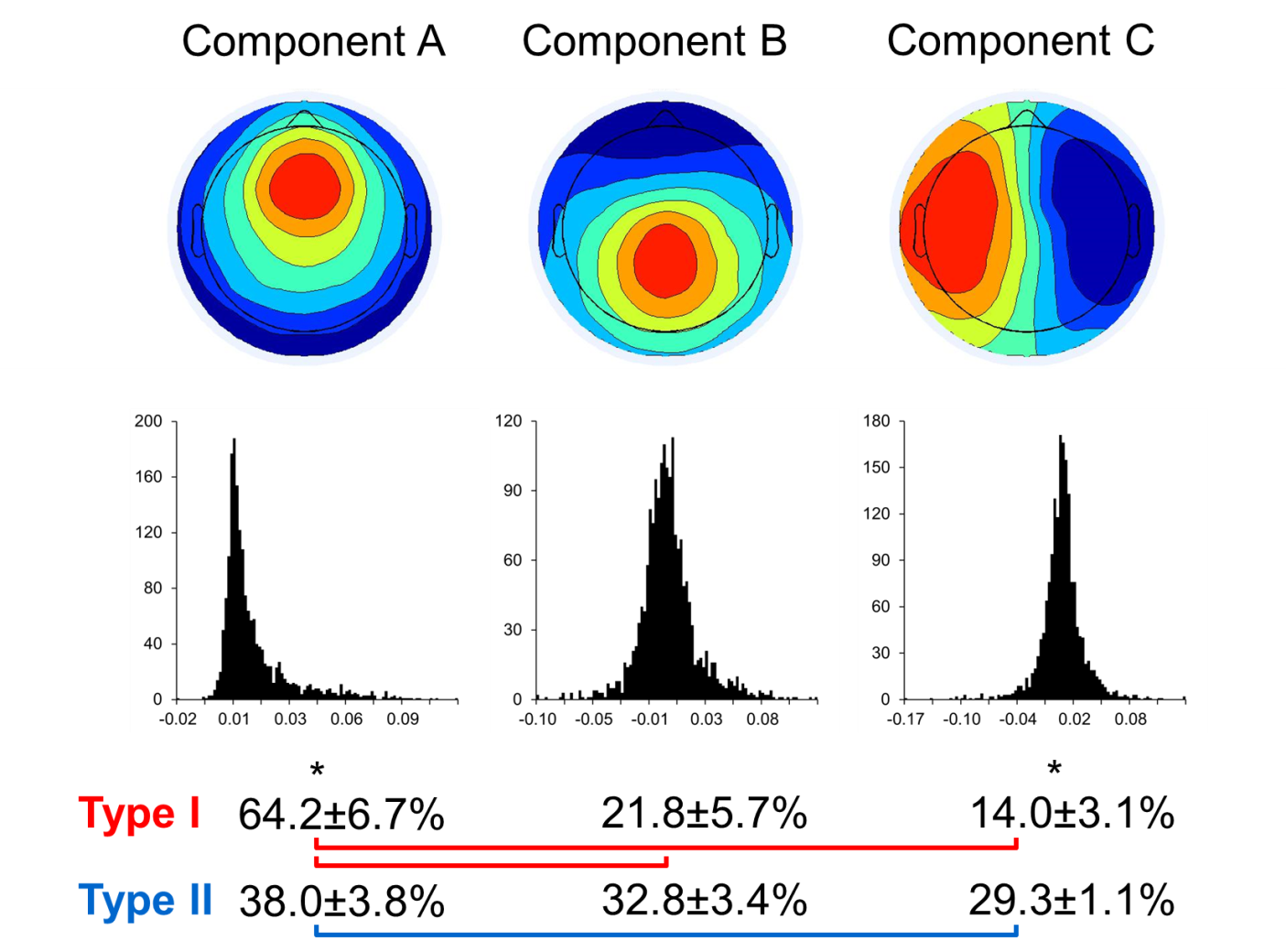


***Figure S4****. The scalp involvement of slow waves can be reduced to three main components (plots and relative weight distributions are obtained from one representative subject). Graphs in mid line depict the ‘contribution’ (relative weight) of individual slow waves to each component. Component A (~69%) is characterized by a distributed involvement peaking over fronto-central areas. Most type I slow waves (>60%) fall into this class. Component B (~18%) shows a dissociation between a central-posterior and a frontal involvement. Component C (~7%) presents a dissociation between a left and a right involvement at scalp level. Type II slow waves showed a similar distribution in all three classes, although the lateralized involvement was less common than the frontal one. Horizontal colored lines indicate significant differences at p < 0.05. * marks significant difference between type I and type II slow waves.*
